# Afferent limb percutaneous jejunostomy to treat pancreatic leak after pancreaticoduodenectomy: a case report

**DOI:** 10.1093/jscr/rjaf930

**Published:** 2025-11-19

**Authors:** Carolina Orsi, Diana Wu, Hishaam Ismael

**Affiliations:** Department of Graduate Medical Education, General Surgery, The University of Texas at Tyler Health Science Center, 11937 US HWY 271, Tyler, TX 75708, United States; Department of Graduate Medical Education, General Surgery, The University of Texas at Tyler Health Science Center, 11937 US HWY 271, Tyler, TX 75708, United States; Department of Surgery, The University of Texas Health Science Center at Tyler, 11937 US HWY 271, Tyler, TX 75708, United States

**Keywords:** pancreatic fistula, percutaneous jejunostomy, pancreaticoduodenectomy, interventional radiology, afferent loop syndrome

## Abstract

Although the mortality after a pancreaticoduodenectomy (PD) is low, it increases with post-operative pancreatic leaks. In this case, a 76-year-old male with pancreatic head adenocarcinoma after successful PD developed a 6 cm fluid collection by the pancreaticojejunostomy (PJ). Interventional radiology proceeded with percutaneous drainage. Unfortunately, the drain color changed from purulent to bilious. A contrast study confirmed the drain’s position within the bowel lumen of the afferent limb. Although not the original intent, the patient progressed. Few cases describe percutaneous drainage of the blind end of the afferent limb. Here, the patient’s chronic bowel dysmotility contributed to increased afferent limb pressures, jeopardizing the PJ. Placement of the intra-luminal drain allowed for source control and decompression that promoted pancreatic leak healing. Hence, percutaneous jejunostomy of the blind jejunal end after PD can serve as a minimally invasive technique for pancreatic leaks secondary to increased pressures within the afferent limb.

## Introduction

Pancreatic fistulas are one of the most harmful complications after a Whipple procedure, consisting of up to 26% of post-operative complications [1]. Often the first signs of a pancreatic leak include elevated drain amylase levels, with severe cases leading to fever development, tachycardia, and increased abdominal pain that may warrant the need for repeated laparotomy [1]. However, these repeat surgeries often come with high morbidity and mortality. Thus, further minimally invasive approaches have been trialed to allow effective and nonoperative treatment. Such modalities include endoscopic or percutaneous, transjejunal drainage techniques that are highly specialized [2, 3]. In this case report, we present a patient after pancreaticoduodenectomy (PD) that was complicated by a pancreatic leak secondary to increased afferent limb pressures from underlying enteric dysmotility. He was successfully treated with percutaneous jejunostomy of the afferent limb, which allowed decompression and pancreatic fistula resolution.

## Case presentation

**Figure 1:**
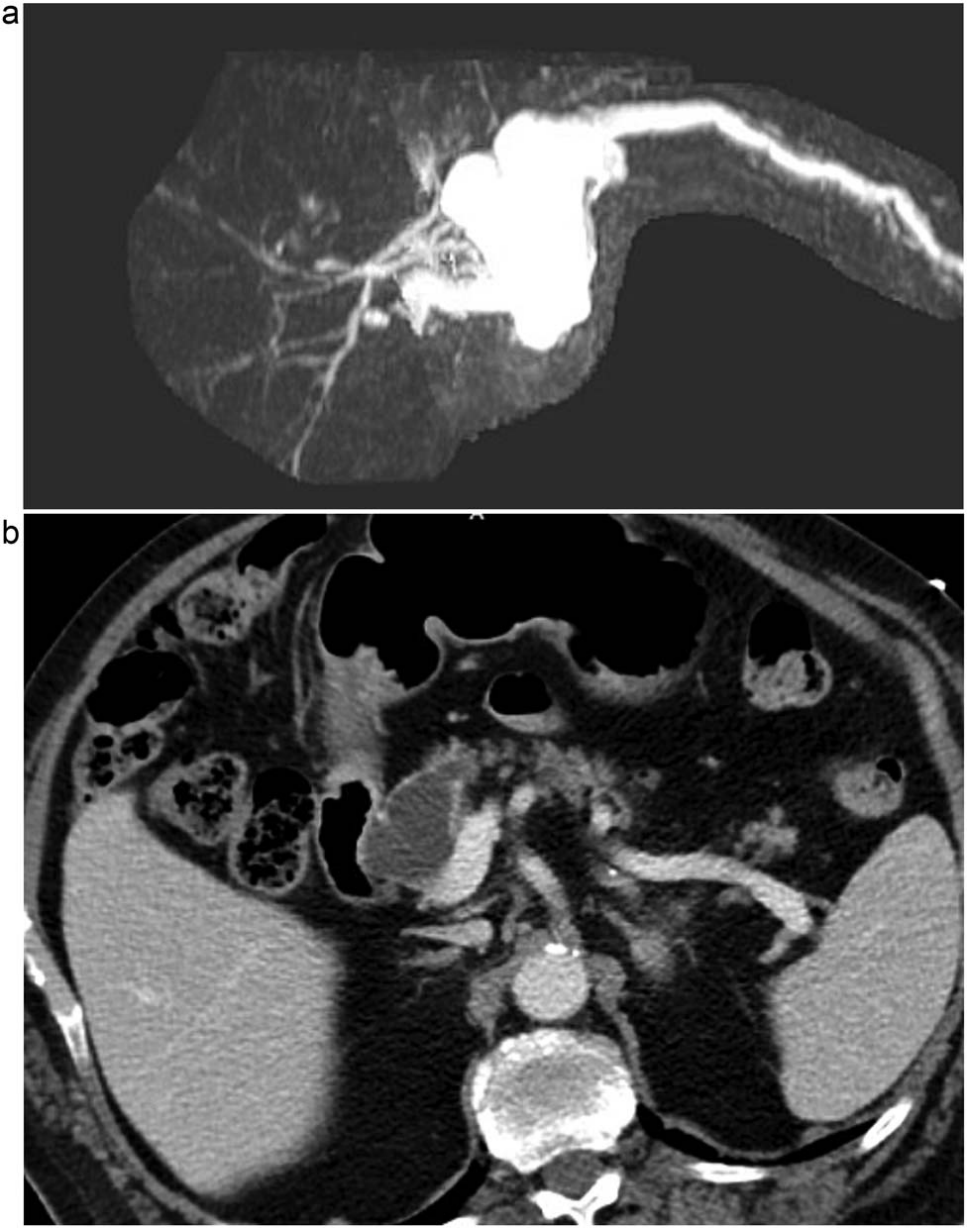
(a) MRI/MRCP imaging of the cystic lesion of the pancreatic head with involvement of the main pancreatic duct. (b) CT imaging illustrating the main duct IPMN at the head of the pancreas.

A 76-year-old male presented initially to the emergency department with concerns of chest pain. His medical history included mild dementia, CHF, aortic stenosis, hypertension, hyperlipidemia, CKD, GERD, and chronic constipation without prior abdominal surgeries. His cardiac workup was negative, however, a CT of his abdomen demonstrated concerns for pancreatic duct dilation. Additionally, MRI evaluation demonstrated a cystic mass at the head of the pancreas that communicated with the main pancreatic duct (see Fig. 1). Initial laboratory values included lipase of 711, CEA of 13, CA19-9 of 11, and normal liver function tests without hyperbilirubinemia.

**Figure 2:**
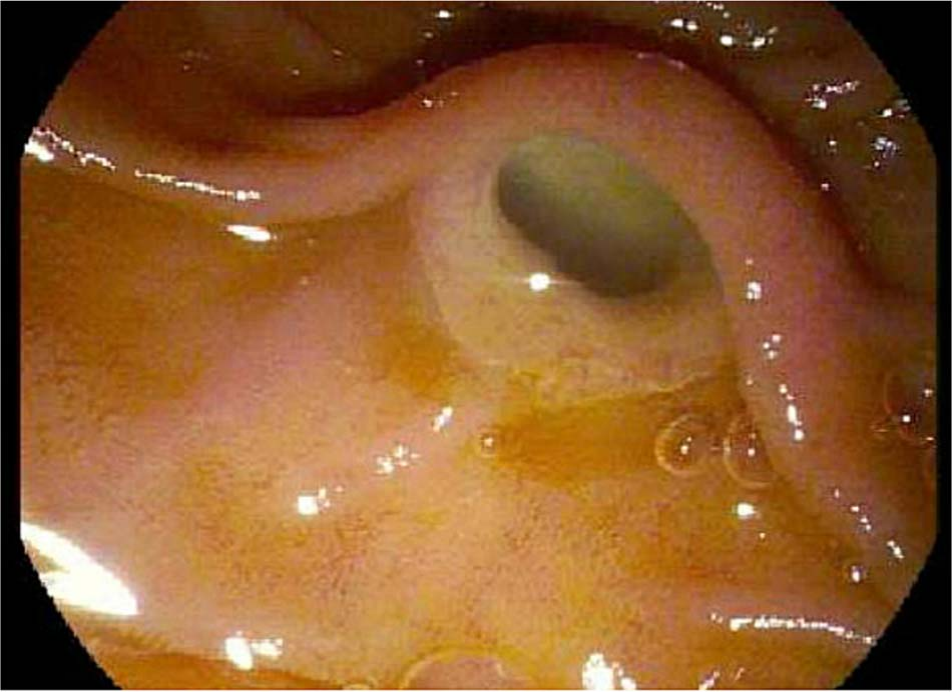
Endoscopic evaluation and classic “fish mouth” appearance of the ampulla.

During the patient’s pre-operative evaluation, an EGD and EUS were performed that revealed a classic “fish mouth” appearance of the ampulla and confirmed the presence of a 3.5 × 3.0 cm cystic lesion at the pancreatic head (see Fig. 2). With findings concerning for a main duct intraductal papillary mucinous neoplasm (IPMN) that appeared resectable, the patient proceeded to the operative room for PD.

Intra-operatively, no carcinomatosis was appreciated. The pancreas was noted to be soft in texture. The pancreaticojejunostomy (PJ) was created in the standard fashion, utilizing the duct-to-mucosa, modified Blumgart technique that was supplemented with pledgets. A Braun enteroenterostomy was also completed in anticipation of potential delayed gastric emptying. Two Jackson–Pratt (JP) drains were placed. Surgical pathology confirmed a low-grade, IPMN with negative margins and a negative lymph node yield.

**Figure 3:**
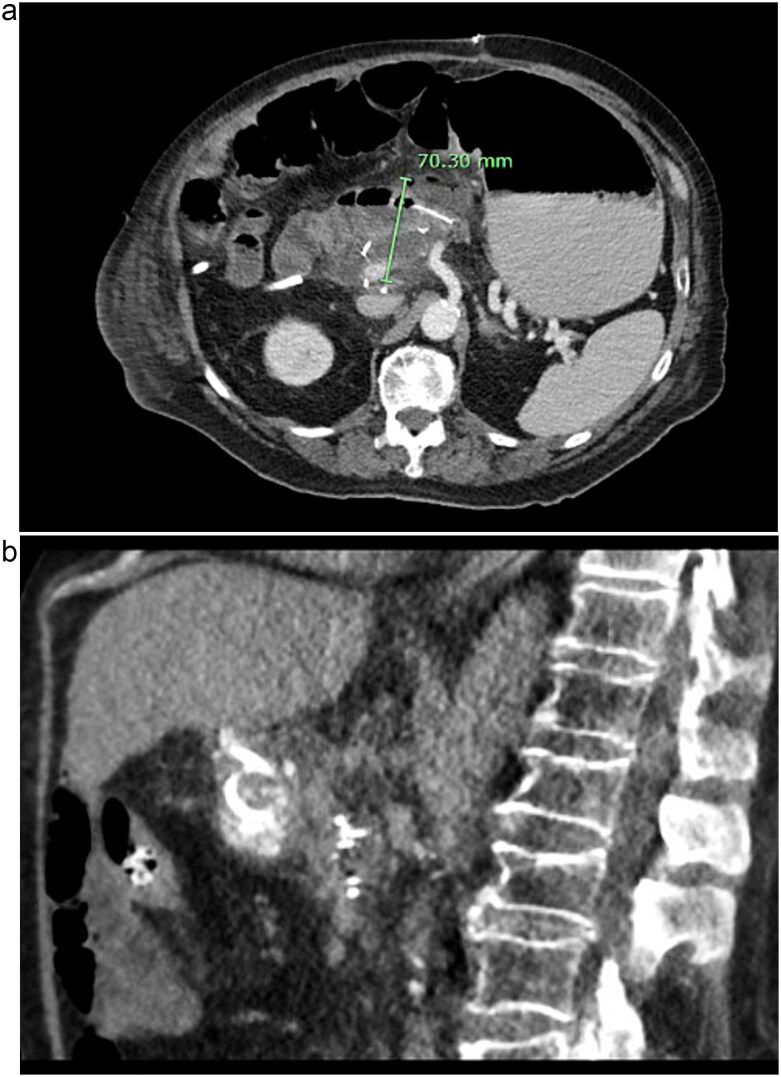
CT images of this patient’s post-operative pancreatic fistula (a) prior to IR guided intervention; (b) after percutaneous jejunostomy that was confirmed to rest within the blind jejunal end of the afferent loop after pancreaticoduodenectomy.

Post-operatively, NGT removal was delayed to post-op day (POD) 3 due to persistently high output. The patient required daily enemas, and his first bowel movement was on POD 7. He was gradually advanced to a low-fat regular diet, while JP drains remained serosanguinous with low drain amylase levels. Unfortunately, he developed a fever of 102.5°F on POD 9 and sudden onset abdominal pain. CT imaging showed a 6 cm fluid collection posterior to the PJ, a persistently dilated stomach, and a high colonic stool burden. An NGT was replaced and total parenteral nutrition initiated. The patient then underwent IR guided aspiration and drainage of the fluid collection within 24 h of symptom onset. After percutaneous drainage, the patient improved clinically. However, ~12 h after drain placement, the drainage changed from purulent to bilious in color. Repeat CT imaging demonstrated that the IR drain had ultimately been positioned within the lumen of the blind end of the afferent limb (see Fig. 3).

Nonetheless, the patient continued to improve and was successfully discharged on POD 18 with all drains in place. Clinic follow up entailed removing both surgical drains and capping the IR drain 3 months after discharge. The IR drain was then completely removed 1 month later, and he continues to do well.

## Discussion

As described previously, pancreatic fistulas after PD have severe clinical impacts on patient morbidity and mortality [4] and therefore systematic methods should be undertaken to reduce this risk. Although pancreatic duct size and gland texture influence the potential of pancreatic fistulas occurring [5], other factors may also contribute and emphasizes the importance of thorough pre-operative evaluation.

This patient’s history of chronic, suboptimal gut motility likely contributed to increased back pressures within his afferent limb that jeopardized his PJ. With his multiple comorbidities including CHF, CKD, aortic stenosis, and dementia, a minimally invasive approach to fistula management was preferred. This coincides with the current literature, in which 90% of pancreatic fistulas are treated successfully with the conservative approach and that surgical intervention serves as the last resort or with development of peritonitis [6]. Therefore, IR was appropriately consulted for percutaneous drainage under image-guidance.

Initially, the IR placed drain was successful in obtaining source control. However, the drain’s position was ultimately found to be within the blind jejunal end of the afferent limb. Although not the intended location, its intra-luminal presence adjacent to the PJ inevitably allowed decompression of the afferent limb. This promoted source control, sepsis resolution, and eventual pancreatic fistula healing without requiring high-risk operative re-intervention.

Scarce literature exists regarding the technique described above. In a 2014 study by Sato *et al*., percutaneous drainage of the blind end of the jejunal limb was performed in the setting of afferent loop syndrome, in which seven out of eight patients experienced relief and adequate pancreatic fistula resolution [7]. Additionally, Inaba *et al*. depicts a successful, yet highly specialized, approach that involves percutaneous, transjejunal drainage with simultaneous pancreatic duct cannulation for fistula management [8]. Our report reinforces the safety of percutaneous jejunostomy decompression of the afferent limb in the setting of pancreatic leaks after PD. Most notably, it also conveys its utility in the setting of poor bowel transit that may lead to higher afferent limb pressures and pancreatic leaks postoperatively.

While previous teaching may have emphasized operative interventions such as surgical drainage, aggressive suturing, anastomotic disconnection, or salvage total pancreatectomy [6], a significant decrease in morbidity is associated with minimally invasive techniques. While further investigation on the role of gut motility is required to correlate its effect on pancreatic fistula rates, this case highlights the influence of increased afferent limb pressures upon PJ anastomoses. Likewise, we illustrate the effectiveness of the percutaneous jejunostomy within the afferent limb for decompression and pancreatic fistula management. Thus, this report brings a unique, relevant, and safe method to treat pancreatic leaks after PD, especially in high-risk patients with high afferent limb pressures.
